# Community centred co-design methodology for designing and implementing socio-behavioural interventions to counter COVID-19 related misinformation among marginalized population living in the squatter settlements of Karachi, Pakistan: a methodology paper

**DOI:** 10.1186/s12919-023-00265-y

**Published:** 2023-07-12

**Authors:** Rubina Qasim, Waqas Ahmed Farooqui, Atiya Rahman, Rukhsana Haroon, Madiha Saleem, Muhammad Rafique, Fiza Noor, Afifa Ghani, Muhammad Yaqoob, Uday Narayan Yadav, Mohammad T. Yousafzai

**Affiliations:** 1 Dow Institute of Nursing & Midwifery, Dow University of Health Sciences, Karachi, Pakistan; 2 School of Public Health, Dow University of Health Sciences, Karachi, Pakistan; 3 Indus College of Medical Technology & Allied Health, The Indus Hospital, Karachi, Pakistan; 4 Indus College of Nursing & Midwifery, The Indus Hospital, Karachi, Pakistan; 5 Dept. of Community Health Sciences, Aga Khan University, Karachi, Pakistan; 6 Centre for Primary Health Care and Equity, University of New South Wales, Sydney, Australia; 7 National Centre for Epidemiology and Population Health, The Australian National University, ACT, Canberra, Australia; 8 Dept. of Pediatrics & Child Health, Aga Khan University, Karachi, Pakistan; 9 The Kirby Institute, University of New South Wales, Sydney, NSW Australia

**Keywords:** Co-design, Design thinking, Misinformation, Community-centred, Vaccine acceptance, COVID-19

## Abstract

**Background:**

Misinformation regarding COVID-19 pandemic and vaccination is damaging COVID-19 vaccine trust and acceptance in Low- and Middle-Income Countries (LMIC). Identification of misinformation and designing locally acceptable solutions are needed to improve COVID-19 vaccine acceptance. This study aimed to utilize community-led co-design methodology to evaluate misinformation regarding COVID-19 and develop contextual interventions to address misinformation in a marginalized peri urban slum communities of Landhi town Karachi, Pakistan.

**Methods:**

This study was conducted between January and December 2021, in marginalized peri-urban slum dwellers of Muslimabad Colony, Landhi Town Karachi, Pakistan. We used a community-centred co-design methodology embedded within mixed study design to identify misinformation, co-design, test and implement locally acceptable solutions. The co-design methodology involved five stages of the design thinking model: (1) Empathize, (2) Define, (3) Ideate, (4) Prototype, and (5) Test. The project involved active engagement and participation of wide range of stakeholders and community beneficiaries (end users) including local EPI vaccinators, informal healthcare workers, religious leaders (male and female), schoolteachers (male and female), local government representatives, community leaders, housewives, youth, and general population. To develop a trusting relationship, and understand local culture, values, practices, and traditions, we allowed one month of observation period (observe, engage, watch, and listen) in the beginning, followed by door-to-door survey along with focus group discussions (FGD) and in-depth interviews (IDI) at baseline. Co-design workshops (separate for male and female) were conducted at each stage of co-design methodology to design and test locally acceptable solutions.

**Conclusion:**

Community-centred co-design methodology was not only successful in designing, testing, and evaluating locally acceptable solutions but it also actively engaged and empowered the marginalized population living in peri urban slum communities of Karachi, Pakistan.

## Background

Misinformation including antivaxx movements regarding vaccines date back to the era of Jenner however, with the widespread use of internet, smartphones and different social media platforms, misinformation can easily and rapidly become viral creating mistrust on vaccines [1], pharmaceutical companies and public health institutions [2, 3]. More recently the spread of misinformation, rumours, and sensationalized stories including conspiracy theories have been a major concern during Zika, Ebola and COVID-19 outbreaks [4–10].

Pakistan is one of the low middle-income countries where immunization rates have always been suboptimum [11]. The country is one of the remaining two countries where polio is still endemic. Vaccine hesitancy and refusals due to misinformation, and various conspiracy theories are widespread [12, 13]. The literacy rate is extremely low among both men and women [14], providing fertile ground for all misinformation and rumours related to vaccinations, damaging trust on vaccines, institutions and healthcare providers [15–17].

It is recognised that globally there is scarcity of evidence in addressing the misinformation, rumours and conspiracies related to vaccination including COVID-19 [18, 19]. People centred locally acceptable strategies with complete ownership by the community, relevant stakeholders and with minimum involvement of the outsiders are required to educate, correct misinformation and address myths and misconceptions regarding COVID-19 infection and its related vaccination in Pakistan [18–21]. A relatively novel co-design method that involve a process of collaborative design thinking where diverse people jointly explore and define a problem, and design and evaluate mutually agreed solutions for the benefits of end users seems viable to explore and address vaccine related misinformation however, this method has never been described and tested empirically [22–25]. This study aimed to utilize community-led co-design methodology to evaluate misinformation regarding COVID-19 and develop contextual interventions to address misinformation in a marginalized peri urban slum communities of Landhi town Karachi, Pakistan.

## Methods

### Study setting

This study was conducted from January to December 2021, among highly marginalized population living in peri-urban slum communities of Landhi Town Karachi, Pakistan. Landhi is one of the 18 towns in Karachi, which is a densely populated predominantly peri-urban town with a population of 553,665 [26]. There are a total of 12 union councils (union council is the smallest administrative unit in Pakistan) located in Landhi town Karachi. Most of the union councils in Landhi are densely populated informal settlements composed of Pashtu speaking impoverished population. These informal settlements are characterized by substandard housing, squalor and lack essential basic health and education facilities. These population predominantly avail health from the first level care facilities (clinics), majority (> 90%) run by unqualified informal healthcare workers [27]. Lack of education, basic health infrastructure and extreme poverty along with widespread misinformation, myths, rumours, and fears regarding vaccination in general put these informal settlements at higher risk of infectious diseases. For this study, two union councils (Muslimabad and Muzaffarabad colony in Landhi town Karachi) with estimated population of about 100,000 to 150,000 were targeted.

### Study population

We involved a wide range of local stakeholders such as different community beneficiaries and influencers (pregnant women, unmarried women, elderly women, female teachers, *Aalima* (female religious teacher), female healthcare provider, housewife, male teacher, male healthcare provider, male EPI vaccinator, local informal healthcare workers, religious leaders/masjid imams, shopkeepers, street vendors, youth, community leaders, and union council chairmen (local government representative).

### Study design

**Fig. 1:**
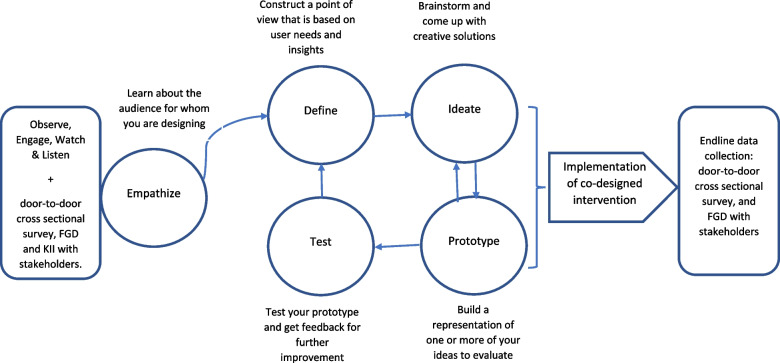
Study design involving mixed methods embedded into the co-design process

This study involved baseline data collection using mixed methods design followed by co-design process to develop contextual interventions (Fig. 1). The co-design methodology involved five stages of the design thinking model proposed by the Hasso-Plattner institute of Design [28, 29]. Each step of the co-design method is explained below.

1. Empathize

Empathy is necessary to understand the problem (what the beneficiaries think and feel? what they need?) so that it could be solved more promptly and accurately. This stage also involves developing rapport with the community and strong engagement with local stakeholders. To develop a rapport, trust, and good working relationship with the community, we hired all the study staff from the same community. Furthermore, to understand local norms, culture, traditions, thinking, the study allowed one month in the beginning to observe, feel, seek stories, and connect with people, community influencers and beneficiaries. This one-month time to observe, engage, watch, and listen assisted in developing strong rapport and trust which was essential for the success of co-design methodology. This period was followed by an active data collection involving both door-to-door cross sectional survey and formative research using focus group discussion (FGD) and key informant interviews (KII) with community influencers. We used a grounded theory approach as this flexible methodological approach allows the involvement of a different group of participants to understand the phenomenon that is little known [19]. This process helped us to observe their behaviour, approaches and underlying factors which influenced their perceptions.

2. Define

In this stage we unpacked empathy into needs and insights and derived an actionable problem statement. We analysed the baseline survey and formative data and derived an actionable problem statement. A separate one day each male and female co-design workshops were conducted where 15 to 20 participants and stakeholders from the community were asked to draw a self-portrait and to briefly describe what aspects of their experiences and strengths could be considered relevant to meet the problems identified. With the help of the community and stakeholders, problems were categorized at the level of Individual/family, Community/society, and service level. Table 1 illustrates the actionable problems at different level.

**Table 1:** Actionable problem statements at different level

Level of Action	Key actionable problem statements
Individual/family level	- Poor health literacy resulting in susceptibility to rumours & mis or disinformation - Lack of females’ involvement in decision-making (poor female empowerment) - Lack of trust in government hospitals and doctors, considered them as party in global COVID-19 hoax - Inherently vaccine hesitant population with hardcore refusal for polio
Community/society level	- Informal healthcare workers as major providers of health - Faith based trust (more trust in religious leaders), strong influence of religious leaders in the community - School teachers and schools influential and respected by the community where literacy is less than 50% - Poverty, lack of civic facilities and healthcare by the govt created lack of trust and negative feelings against the government
Service level	- No COVID-19 vaccination centre in proximity - WhatsApp, YouTube, and Facebook as major source of acquiring information - Local informal healthcare providers, religious leaders, schoolteachers, and school going youth considered as knowledgeable regarding COVID-19 pandemic & vaccination

3. Ideate

Ideate is the mode and ideation are a process where within the context of the problem statement, the team generates a radical design. The goal of ideation is to explore a wide solution. In this process, we allowed ourselves in divergent thinking (brainstorming and mind-mapping exercises) followed by convergent thinking to synthesize (i.e., refine and integrate) collections of ideas into a cohesive viable concept. The interventions developed at this stage were guided by evidence and the theory of change or action. This entire process resulted in the development of key solutions or interventions to address the misinformation related to COVID-19 and improve COVID-19 vaccine acceptance. A co-design workshop was conducted where the derived interventions were shared with a bigger team of stakeholders that included policy makers, general population, and religious leaders (*n* = 20) to get more insight. Table 2 provides the list of solutions along with theory of change.

**Table 2:** The list of ideated solutions and theory of change

Level of Intervention	Small group ideas	Theory of change
School level	Awareness campaign in schools located within the community Distribution of pamphlets with clear/correct messages regarding COVID-19 and COVID-19 pandemic, preventive measures, masking practice, role modelling by the volunteers and involvement of schoolteachers and secondary level students. Child to child approach and child to parents/family approach where every child/student will educate one child and/or family member	Schools as institutions and school children and teachers are highly trusted and respected in the community. They are the potent source of spreading misinformation. Improving their health literacy related to COVID-19 and better equip them with correct knowledge will not only block the spread of misinformation further but it will also decrease their susceptibility to future mis or disinformation. They are also highly trusted mode of delivery of correct information in this community
Madrasa (an educational institution offering Islamic education) arranged separately for male and female	Awareness campaign regarding how to take preventive measures against COVID-19, how to get register for COVID-19 vaccination and information to counter widespread misinformation Female and male religious scholars (Aalima and Aalim) should be trained and actively involved during these campaigns	Madrasas are considered sacred institutions which hold a special place in Pashtun society living in the target community. Both male and female religious teachers associated with these Madrasas and students of these Madrasas are highly respected in society and trusted for their words. Therefore, specifically, designed for female religious students who do not have access to radio, television or newspapers, smartphone, or any social media, in contrast to males Awareness campaign will spread key messages focusing on hygiene and infection prevention, including hand washing using soap, social distancing, and identify widespread misinformation regarding COVID 19 and COVID 19 vaccination. This will also empower female population to register themselves for COVID-19 vaccination
Local clinics and maternity home	Active involvement of local informal and formal healthcare providers in co-design workshops, satisfying their concerns, queries, and misconceptions, providing them relevant health literature regarding COVID-19 pandemic and COVID-19 vaccination and sensitizing them to the widespread mis and disinformation. Distribution of pamphlets, flyers through their clinics and helping them counsel their patients regarding COVID-19 pandemic and vaccination if anyone seek their advice	Informal healthcare workers are the major service providers in target community. They are highly regarded, trusted, and consulted by the community regarding decisions of vaccination and/or preventive measures. Our baseline data also demonstrated that these informal healthcare workers lack optimum healthcare literacy and in fact they are also vulnerable to mis information and misconceptions regarding COVID-19. They are also disengaged in vaccination programs. Active involvement of this large informal force and better equipping them with correct information and health literacy will improve the community confidence in vaccination and debunk misinformation
Mosque	Masjid imams (religious leaders leading prayers in mosques) are the highly trusted people with a large following in target community. Their issues, concerns, and queries regarding the COVID pandemic should be addressed by mutual discussion and respect of their opinion. They will be satisfied and assured that by participating in this project they are not committing any sin but instead doing the public good to fight fake news. These Masjid imams should be empowered to tailor messages considering holy Quran e.g., a verse from a holy Quran which says “do NOT spread fake news” for the community Megaphones/ loudspeaker and posters will be used to explain COVID-19 and to debunk myths and conspiracy associated with the virus, and COVID-19 vaccination	Masjid imams are the highly influential people in the target community and especially in the Pashtun population. Our baseline data showed they are highly trusted by the community, and they are considered an authority in every subject. People refer to them to seek their guidance regarding getting their children vaccinated. Empowering them with correct information and actively involving them to tailor messages which are acceptable from the religious point of view (and not contradictory to Islamic Sharia) and delivering those messages through them will dispel the misinformation and foster an environment of trust for vaccine acceptance
Role of youth in Harnessing social media	Creation of youth WhatsApp group in each neighbourhood. Since an enormous number of smartphones is used by youth, they are highly active in social media for communication with friends and family They should be used to circulate educational videos which will be created by local actors (nursing students hired from same community and trained by research team) working in different hospitals	Mis and disinformation thrive in social media, where literacy rate is low and when access to digital and main media is narrow These educational videos created by nursing students in local language will be circulated through WhatsApp, run by youth from local community. These youth will circulate the videos among friends, family, relative and help in discerning the truth Promote positive and hopeful stories which will increase positive behaviour and attitude regarding COVID-19 pandemic, preventive measures, and vaccination uptake. Further, these groups will also respond to any viral misinformation or disinformation regarding COVID-19
Door-to-door visits	Door-to-door visits with the distribution of pamphlets. Research field staff along with trained volunteers will distribute pamphlets keeping in mind the very fact that this community is informationally vulnerable to place-based mis and disinformation especially with limited quality information sources at local levels, so it is exceedingly easy to misrepresent local-level events and conditions and provide fake evidence or a sense of momentum for broader disinformation narratives	This will help to control the spread of misinformation Shed anti vaccination perception Help in positive static attitudes for vaccine acceptance
Social Media	use of social media such as Facebook page, YouTube channel and twitter where members from the community can be encouraged to follow	Social Media platforms will facilitate an informational environment, where access to mass media and digital media is limited
Community suburbs	Conducting community concert type health Mela where loudspeakers can be used to disseminate correct information	Health mela with active involvement of community stakeholders, study volunteers and research team will use loudspeakers and music to attract local community and disseminate correct messages to address fear, misconceptions, rumours, and misinformation regarding COVID-19 and vaccination. Further to also improve trust in vaccines

4. Prototype

In this stage, the broader list of ideated solutions was validated for its conceptualization and appropriateness and then refined. The main purpose of this stage was to refine the interventions and come up with a single most suitable prototype/intervention and delivery strategy required for the testing or implementation phase. This stage included a preliminary consultative meeting with local stakeholders and community beneficiaries that give the opportunity to every participant to discuss the appropriateness and use of the proposed intervention strategies.

Due to lockdown, two virtual (zoom) consultative meetings of one hour each for male and female participants were organized. The female stakeholders’ participants included female schoolteachers, religious female teachers, informal healthcare providers (lady health visitors, traditional birth attendants, and/or unqualified or licensed lady doctors). Few housewives also participated. Altogether, only 15 female and 20 male participants attended these two virtual sessions. Based on the discussions at this phase, a single mature package of intervention involving four different strategies was finalized for testing and evaluation. The intervention package is listed below.

A School based approach to disseminate correct contextual information and knowledge to the school children and their teachers and in turn these children and teachers will disseminate that information to their families.
B Masjid (mosque) based approach to disseminate the same contextual messages to the masjid imams (religious leaders) and they convey the message to their followers.
C Healthcare providers-based approach to actively involve and educate informal healthcare providers who have clinics and maternity homes in this neighbourhood. These healthcare providers will be provided with correct information, and they will further communicate and educate their patients and attendants.
D Youth based approach by creating WhatsApp groups of youth from each neighbourhood and they are provided with short video clips, messages, and text to disseminate and correct misinformation. The research coordinator and research assistants will closely follow and moderate these groups and if there is any misinformation circulating through social media, the rebuttal will be circulated through these WhatsApp groups.

5 Testing

This phase involved the implementation of the designed intervention package in target population and its evaluation/feedback. The test mode was an iterative mode where the intervention package was evaluated and refined based on users’ feedback. Testing was done by involving four secondary level schools, two madrassahs, two WhatsApp groups for youths, ten religious’ leaders, and about twenty informal healthcare workers from target communities. Details about the infographics, video clips and communication messages along with the detailed findings are going to be published in a separate paper. Findings from the formative research are already published [20]. Ethical approval was obtained from the institutional review board of Dow University of Health Sciences, Karachi, Pakistan.

## Discussion

This study involved community cantered co-design methodology embedded within a mixed methods study to explore misinformation regarding COVID-19 pandemic and COVID-19 vaccination in an ultra-marginalized peri urban slum community of Karachi, Pakistan. A co-design process works on the principles of social democracy, equity, identifying and understanding problems and finding mutually agreed solutions with and for the benefits of the end-users [23–25]. It involves active engagement of the community and all stakeholders starting from exploring problems, identifying priorities, ideating, and finalizing locally acceptable solutions tailored to the local needs and implementing those solutions with and for the people for whom it is designed [25, 30]. Meaningful and active involvement of community and stakeholders with different backgrounds is important for the success of any population-based intervention program [24, 31]. Interventions developed through co-design process has better acceptance among the end-users, can be easily implemented anywhere with little input from the outsiders, and with the involvement of stakeholders such as EPI program, religious leaders, informal healthcare workers and community leaders, long term sustainability to counter future misinformation and rumours damaging trust on vaccination is possible.

Unlike traditional research that has a top-down approach, resulting in poor response rate, community acceptance and no empowerment of the end users, co-design method operates on the bottom-up approach where community, local stakeholders, and their empowerment rest in the centre of the whole process [32]. The success of co-design method depends on an earlier engagement of the stakeholders, winning their trust, and involving them at each step, starting from planning to implementation and evaluation [33]. Likewise, co-design process shifts the intervention or solution design away from an expert-driven process towards end-users [32]. Active involvement of end users, enabling them to perceive or define their problem, understand how the outsiders perceive their problem, and empower them to jointly develop solutions, are important attributes of a successful co-design process [22].

To the best of our knowledge, co-design methodology has never been applied before in Pakistan especially in marginalized peri urban population with poor literacy and health utilization. Therefore, successful implementation of this project provided important evidence that poor literacy among target population should not be a hindrance in using the community centred co-design methodology for designing, implementing, and evaluating health interventions in similar settings.

As co-design method operates on the principles of equity and social democracy, we recommend equal involvement of the female population and female stakeholders e.g., female schoolteachers, informal healthcare workers, female religious teachers, youth, and housewives. Based on the initial observation phase (observe, engage, watch, and listen), we found that females in our target population follow strict veiling and seclusion. Moreover, females were reluctant to participate and did not perceive themselves suitable for useful contribution and decision-making process. Thus, we arranged separate co-design workshops for both male and female population with female workshops facilitated by female staff only. Also, we recommend understanding and respecting the local culture, practices, and traditions to develop rapport and trust which is essential for co-design methodology. In addition, to empower community to jointly learn, identify, design and evaluate solutions for their problems, we recommend facilitators must help them aware of their thoughts, feelings, responsibilities and interests at the outset [22]. Lastly, some of the participants and stakeholders dominate each step of co-design process without respecting the opinion of other participants and /or giving them opportunity to speak or give their input and hence, we recommend facilitators must be vigilant to avoid such situation. Participants who are less vocal must be encouraged and given equal opportunity to participate and contribute to the discussion and decision-making process.

## Challenges and lessons learnt

Several challenges were faced during the field operation including difficulty finding female research assistants from target community, resistance to photograph or audio-record female stakeholders and community beneficiaries, lack of willingness for virtual meetings and workshops as opposed to face-to-face meetings and scepticism to cooperate during the baseline data collection. To address these problems, we hired female polio workers and Pashtu speaking female BSc Nursing students from target communities for data collection and co-design workshops. Likewise, photographs of female stakeholders and participants were taken after informed consent, not revealing the faces of the female population. Activities involving audio recordings such as in-depth interviews and focus group discussions were delayed until the community gain full trust, confidence, and good working relationship was established with the field teams. Stakeholders and most of the community beneficiaries were unavailable for six hours of co-design workshops on the working days and hence all co-design workshops were conducted on Sundays. Similarly, zoom calls were held in the night-time based on the comfort of community beneficiaries. Despite challenges, co-design process was successful in engaging community and stakeholders. We learnt that co-design is a successful approach in winning the trust of stakeholders that leads to active engagement of stakeholders, ownership, capacity building and sustainability in marginalized population. Interestingly, following the initiation of the testing phase of the interventions, the target community started visiting our research office asking for COVID-19 vaccination. We observed an increasing demand for the COVID-19 vaccines (which was not available at the local EPI centre and private market). Based on the persistent demand from the community and strong networking and engagement with the local stakeholders from the EPI program and town health officials, a weekly outreach COVID-19 vaccination centre was setup at our research office where target community was provided with free vaccination services.

## Conclusion

Community-centred co-design methodology was not only successful in designing, testing, and evaluating locally acceptable solutions but it also actively engaged and empowered the marginalized population living in peri urban slum communities of Karachi, Pakistan.

## Data Availability

This manuscript is based on methodology only and no data is reported here.
